# Does hydrostatic pressure influence lumpfish (*Cyclopterus lumpus*) heart rate and its response to environmental challenges?

**DOI:** 10.1093/conphys/coab058

**Published:** 2021-07-22

**Authors:** Zoe A Zrini, Rebeccah M Sandrelli, A Kurt Gamperl

**Affiliations:** Department of Ocean Sciences, Memorial University, St. John’s, Newfoundland and Labrador, A1C 5S7, Canada

**Keywords:** Biologgers, heart rate, hydrostatic pressure, hypoxia, lumpfish, temperature

## Abstract

Studies on the effects of environmental changes with increasing depth (e.g. temperature and oxygen level) on fish physiology rarely consider how hydrostatic pressure might influence the observed responses. In this study, lumpfish (*Cyclopterus lumpus*, 200–400 g), which can exhibit vertical migrations of over 100 m daily and can be found at depths of 500 m or more, were implanted with Star-Oddi micro-HRT loggers. Then, their heart rate (*f*_H_) was measured in a pressure chamber when exposed to the following: (i) increasing pressure (up to 80 bar; 800 m in depth) at 10°C or (ii) increasing temperature (12–20°C), decreasing temperature (12 to 4°C) or decreasing oxygen levels (101–55% air saturation at 12°C) in the absence or presence of 80 bar of pressure. Additionally, we determined their *f*_H_ response to chasing and to increasing temperature (to 22°C) at atmospheric pressure. Pressure-induced increases in *f*_H_ (e.g. from 48 to 61 bpm at 12°C) were associated with hyperactivity. The magnitude of the rise in *f*_H_ with temperature was greater in pressure-exposed vs. control fish (i.e. by ~30 bpm vs. 45 bpm between 5°C and 20°C). However, the relative increase (i.e. slope of the relationship) was not different between groups. In contrast, 80 bar of pressure eliminated the small (5 bpm) increase in *f*_H_ when control fish were exposed to hypoxia. Exhaustive exercise and increasing temperature to 22°C resulted in a maximum *f*_H_ of 77 and 81 bpm, respectively. Our research shows that pressure influences the *f*_H_ response to environmental challenges and provides the first evidence that lumpfish have a limited capacity to increase *f*_H_.

## Teaser

There is limited information on how hydrostatic pressure affects the fish heart’s response to other environmental challenges. Exposure to 80 bar of pressure increased the lumpfish’s activity and heart rate and eliminated their heart rate response to moderate hypoxia. However, it did not affect how heart rate changes with temperature.

## Introduction

During vertical migrations to deeper waters, animals experience large changes in environmental conditions such as increases in hydrostatic pressure and reductions in temperature, oxygen (hypoxia) and light (Gross and Jaenicke, 1994; Andrzejaczek *et al*., 2019). However, due to the technical difficulties and high costs of gaining biological information while animals are under pressure (Guerrero *et al*., 2000; Shillito *et al*., 2014), there is still very little known about the physiological responses of fish to changing environmental conditions at depth (Andrzejaczek *et al*., 2019).

The physiological capacity of animals to cope with changes in their environment can inform biologists and managers of a species’ likelihood to be a ‘winner’ or ‘loser’ in the face of ocean warming and the expansion of oxygen minimum zones (Somero, 2010; Cooke *et al.*, 2013). For example, changes in water temperature and oxygen levels have implications for future shifts in the bathymetric distribution of fish (Morris *et al*., 2015; Andrzejaczek *et al*., 2019). Specifically, it is primarily the capacity of the cardiovascular system that limits the depth range of ecologically and economically important species such as tuna and billfishes (Brill *et al.*, 1998). Further, heart rate (*f*_H_), cardiac output and the scope available for *f*_H_ may well determine the survival of fishes when exposed to abiotic and biotic challenges (Eliason *et al.*, 2013; Farrell and Smith, 2017). Thus, it is critical that we learn more about the effects of these interacting variables on fish cardiovascular function.

Early research on the effects of hydrostatic pressure examined the tolerance and behavioural responses of a narrow range of fish species to acute increases in pressure (Brauer *et al*., 1974; Macdonald *et al*., 1987). Since then, the field has focused on the effects of acute and chronic pressure increases on the metabolic response of various fish species (e.g. see Sébert and Barthélémy, 1985a, b; Sébert and Macdonald, 1993; Speers-Roesch *et al*., 2004; Vettier *et al*., 2005, 2006). However, to our knowledge, no studies have measured the physiological response of fish to hypoxia in combination with pressure and very few studies have examined the combined effects of temperature and pressure (Sébert *et al*., 1995a, b; Scaion *et al*., 2008a, b). Further, even less is known about its effects on the cardiovascular system, and the published information is quite variable. For example, some studies indicate that pressure causes tachycardia in fish (Naroska, 1968 (c.f. Sébert and Macdonald, 1993); Sébert and Barthélémy, 1985b), while others report bradycardia (Belaud *et al*., 1976; Pennec *et al.,* 1988), and the mechanisms mediating these effects are not yet known.

The common lumpfish (*Cyclopterus lumpus*) is an ecologically important marine species that is widely distributed on both sides of the Atlantic Ocean (Simpson *et al*., 2016; Powell *et al*., 2017). Further, it is a commercially important species due to the demand for their role as a substitute for sturgeon caviar, and their use as a cleaner fish in the Atlantic salmon (*Salmo salar*) aquaculture industry (Imsland *et al.*, 2014; Powell *et al*., 2018). However, due to overfishing/harvesting, lumpfish have been designated as ‘Threatened’ by the Committee on the Status of Endangered Wildlife in Canada (COSEWIC, 2017). Information on the physiological limits of this species will be important for its future conservation, and its proper management in the roe fishery and in the aquaculture industry. However, there is a limited understanding of the basic physiology of lumpfish and their tolerance to different environmental conditions (Ern *et al*., 2016; Jørgensen *et al*., 2017; Hvas *et al*., 2018; Hvas and Oppedal, 2019).

As mature adults, lumpfish migrate into shallow coastal waters in the spring and summer to reproduce. However, once the young become juveniles, they migrate out to the open ocean (Davenport, 1985). Most pelagic trawl records and video images suggest that lumpfish reside in the upper 60 m of the ocean, but they have also been found at depths up to 1000 m (Blacker, 1983; Rosen and Holst, 2013; Rosen *et al*., 2013). In addition, Kennedy *et al*. (2016) tagged Icelandic lumpfish with biologgers that recorded depth and temperature and they reported that the maximum recorded and extrapolated depth (based on temperature data) were 309 and 498 m, respectively, and that this species regularly engaged in daily vertical migrations of greater than 100 m.

Given that lumpfish can be found at a variety of depths and that data on their cardiovascular physiology and environmental tolerances are lacking, we set out to determine how hydrostatic pressure influences their capacity to deal with other abiotic factors. Star-Oddi micro-HRT loggers have been shown to provide reliable measurements of *f*_H_ in other fish species (Brijs *et al.*, 2019; Zrini and Gamperl, 2021) and would allow us to collect measurements of *f*_H_ in a sealed pressure chamber (IPOCAMP, formally named ‘Incubateur Pressurisé pour l’Observation et la Culture d’Animaux Marins Profonds’). Considering the lack of information on the effects of pressure on the cardiovascular system of fish, we used these two unique pieces of equipment to examine: (i) the *f*_H_ response of lumpfish to increasing hydrostatic pressure up to 80 bar (800 m in depth); (ii) the effect of prior exposure to 80 bar on the post-chase *f*_H_ of lumpfish; and (iii) the *f*_H_ response to decreasing temperature (12 to 5°C), increasing temperature (12 to 20°C) or decreasing oxygen levels (101–55% air saturation at 12°C) in the absence and presence of 80 bar of pressure. The experiment investigating the effect of increased pressure and temperature (which would be unlikely to occur unless fish were in the proximity of hydrothermal vents) was performed to more fully understand how temperature and pressure interact in the control of *f*_H_ in fishes. In addition, we measured the lumpfish’s *f*_H_ response during an acute warming protocol (increase by 2°C h^−1^ up to 22°C) at atmospheric pressure. This latter experiment was performed to determine if the limited maximum *f*_H_ observed for this species in the IPOCAMP was similar to that measured by a standard warming protocol.

## Materials and methods

### Animal husbandry

All work described was approved by the Institutional Animal Care Committee of Memorial University (Protocol ^#^17-95-KG) and followed the standards and guidelines outlined by the Canadian Council on AnimalCare.

The lumpfish used in these studies were reared at the Ocean Science Centre (Memorial University; Newfoundland, Canada) at 6°C. The lumpfish were then transferred to one of two 0.5-m^3^ tanks on 23 April 2018 (*n* = 56), 23 July 2018 (*n* = 22) and 16 January 2019 (*n* = 20) with densities never exceeding 16.8 kg m^−3^. Tanks were supplied with seawater at ~7.5°C and a 14-hour light:10-hour dark photoperiod. The temperature in these tanks was raised at a rate of 0.5°C per day to 10 or 12°C, this difference in temperature due to requirements of concurring projects in our multi-use facility. Lumpfish were held at these temperatures for a minimum of 14 days before use in experiments.

### Logger implantation

The Star-Oddi (Gardabaer, Iceland; see https://www.star-oddi.com) micro-HRT logger (25.4 mm in length, 8.3 mm in diameter, 3.3 g in air) records and stores *f*_H_, electrocardiograms (ECGs) and temperature. These loggers were programmed using the company’s Mercury software prior to implantation.

To prepare the loggers for implantation, 3-O silk suture was tied around the body of the logger and the loggers and surgical equipment were cleaned and sterilized in 70% ethanol. The lumpfish were anaesthetized in seawater containing 0.15 g l^−1^ of tricaine methanesulfonate (MS-222; Skar *et al*., 2017). After losing equilibrium, the fish were moved onto a wetted surgical sponge where their gills were irrigated with flowing and aerated ~10°C seawater containing 0.075 g l^−1^ of MS-222. A 1.5- to 2.0-cm mid-ventral incision was made in the fish’s body wall beginning immediately posterior to the sucker. The logger was then inserted (blunt end first) in a posterior direction and pulled anteriorly to within 0.5 cm of the pericardium using the anchoring suture. A cutting-edge needle was used to pass the suture through the skin to secure the logger to the body wall at the anterior of the incision. Finally, the incision was closed with continuous stitches. One or two suture knots were also attached to the dorsal muscle to allow for the identification of fish in their holdingtank.

### IPOCAMP set-up

Experiments ^#^1 and ^#^2 used IPOCAMP chambers (Autoclave, France; 19 l vessel, 60 cm high by 20 cm in diameter) (Fig. S1). The temperature of the chambers was controlled by a heater/chiller that regulated the temperature of both the in-flowing water and of the glycol jacket that surrounded each chamber. The water flowing into the IPOCAMPs came from a 50-l reservoir in which the oxygen level was controlled by a fibre-optic oxygen probe connected to a Witrox 1 oxygen system equipped with WitroxCTRL software and solenoid valves (Loligo Systems, Denmark). This system regulated the reservoir’s water oxygen content within relatively narrow limits (±2% air saturation) by bubbling air or nitrogen into the reservoir when water oxygen levels reached the lower and upper set points, respectively. These set points were determined by monitoring the oxygen content in the water leaving the chamber, as recorded by a Fibox 3 LCD oxygen metre (PreSens, Germany). A PT1000 probe was used to measure temperature in the reservoir (±0.15°C), and a pipe inspection camera was inserted into one of the view ports in the lid of the IPOCAMP to record the behaviour and activity of the fish during all experiments. Fibre-optic light sources and red filters were inserted into the other two view ports to provide adequate light and to maintain photoperiod.

### Experiment ^#^1: heart rate response to hydrostatic pressure and the fish’s maximum post-exercise heart rate

After implantation, lumpfish (*n* = 14, 0♀: 0♂: 3 immature: 11 not measured, 237.8 ± 5.3 g, 18.1 ± 0.3 cm, mean ± S.E.M.) were returned to their tank for recovery. At 48 hours post-surgery, two fish were transferred to a container with mesh sides (38.7 cm long × 24.8 cm wide × 29.2 cm deep) that was floating in the tank to be fasted for ~66 hours before being transferred to the IPOCAMP. This was necessary as water supplying the IPOCAMP passed through a fine filter that was easily clogged by faecal matter. Then fish, two at a time, were placed on the platforms of an insert that was lowered into the IPOCAMP (Fig. S1) and acclimated to the chamber overnight at 10°C and at 0 bar of pressure.

**Figure 1:**
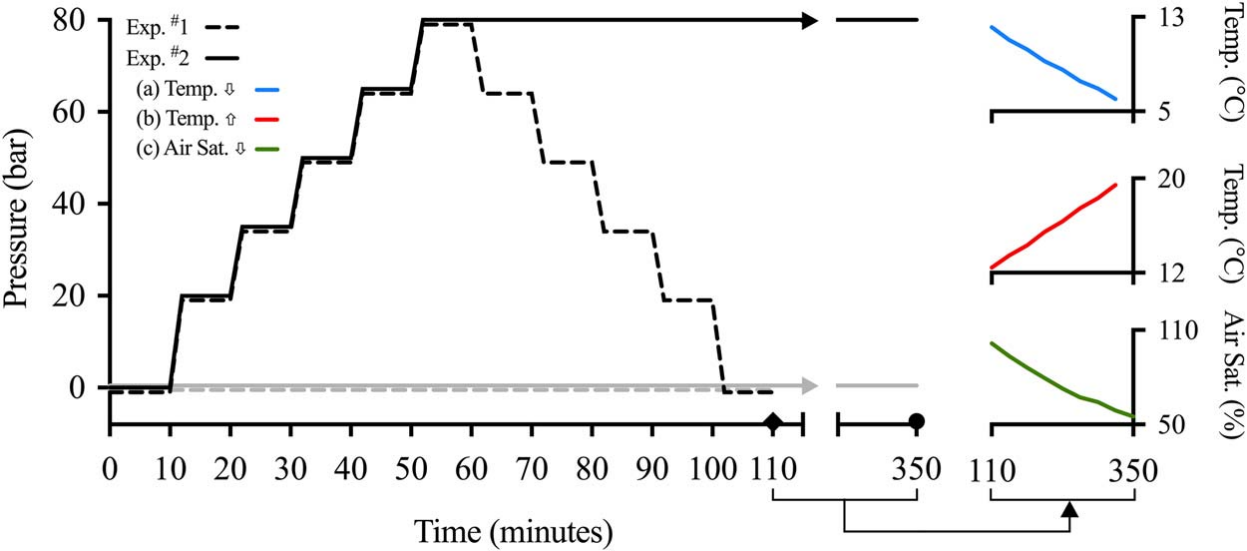
Protocols for compression and/or decompression in Experiment ^#^1 (dotted lines) and ^#^2 (solid lines). In Experiment ^#^1, control lumpfish (grey lines) were held at 0 bar and the pressure-exposed fish (black lines) were compressed to 80 bar in a stepwise protocol and then decompressed to 0 bar using the same protocol (110 total minutes; diamond represents end of experiment). In Experiment ^#^2, control fish were held at 0 bar and pressure-exposed fish were compressed to 80 bar using the stepwise protocol and held at pressure. After 1 hour, control and pressure-exposed fish were exposed to either: (a) decreasing temperature, (b) increasing temperature or (c) decreasing oxygen (340–350 total minutes; circle represents end of experiment).

Immediately following surgery, the pre-programmed micro-HRT loggers saved ECGs and recorded *f*_H_ (at 100 Hz for 6 seconds) and temperature every 4 hours during the recovery period. On the morning following acclimation to the IPOCAMP, the loggers began to save ECGs and record *f*_H_ (100 Hz for 6 seconds) and temperature every 2 minutes. Then, the lumpfish were exposed to 0 pressure (*n* = 6; for use as time-matched controls) or to increasing levels of hydrostatic pressure (*n* = 8). Hydrostatic pressure was initially increased to 20 bar over 2 minutes, then held at this pressure for 8 minutes. Thereafter, pressure was increased to 35, 50, 65 and finally to 80 bar using the same protocol. The lumpfish were then decompressed in the opposite sequence (see Fig. 1 for a schematic representation of this experiment).

Following the experimental protocol, most lumpfish were removed from the IPOCAMP and euthanized. However, four fish from each treatment were given 1 hour in the chamber for the pressure-exposed group to recover. Then, these fish were placed into a tote (75 cm long × 40 cm wide × 45 cm deep, 30 l) and continuously chased with a net for 1.5 minutes. Lumpfish were discouraged from attaching to the tote by constant poking/encouragement with the net. This allowed for an estimation of the fish’s maximum *f*_H_ in response to exercise with and without prior exposure to pressure. However, no fish lost equilibrium during the chase or appeared to reach complete exhaustion, and thus their maximum post-chase *f*_H_ may not have been reached.

### Experiment ^#^2: influence of hydrostatic pressure on heart rate and its response to changes in temperature and hypoxia

Lumpfish were recovered from surgery, fasted and transferred to the IPOCAMP chamber as described in Experiment ^#^1, but at 12°C. The micro-HRT loggers were set to save ECGs and record *f*_H_ (100 Hz for 6 seconds) and temperature every 4 hours on the day of being transferred to the IPOCAMP, every 2 minutes during the increase in pressure and when initially held at 80 bar (i.e. between 8:00 and 10:20 AM) and every 5 minutes for the rest of the duration of the experiment; the latter used to save logger memory and batterylife.

On the morning following acclimation to the IPOCAMP, lumpfish were exposed to increasing pressure to 80 bar and acclimated at this pressure for 1 hour, or maintained at atmospheric pressure (0 bar, control fish) for the same total duration (Fig. 1). The lumpfish were then exposed to one of the following treatments. In the first trial, lumpfish were exposed to decreasing temperature from 12°C to 5°C (*n* = 14, 7♀: 2♂: 3 immature: 2 not measured, 350.1 ± 12.4 g, 21.1 ± 0.2 cm). In the second trial, lumpfish (*n* = 15, 1♀: 0♂: 8 immature: 6 not measured, 404.9 ± 14.1 g, 21.2 ± 0.3 cm) were exposed to increasing temperature from 12 to 20°C (~2°C lower than the previously calculated critical thermal maximum or CT_MAX_ for lumpfish; Ern *et al*., 2016). The rate of temperature change in both trials was ~ 2°C h^−1^. Finally, lumpfish (*n* = 16, 2♀: 6♂: 4 immature: 4 not measured, 435.8 ± 23.9 g, 21.9 ± 0.4 cm) were exposed to decreasing oxygen from 100% to 50% air saturation (~10% air saturation above their P_crit_ calculated at 10°C; Ern *et al*., 2016). Temperature and oxygen were not brought close to the CT_MAX_ or P_crit_ of the lumpfish because they can attach to the platforms with their suckers even when unconscious, making loss of equilibrium difficult to determine (Ern *et al*., 2016). In addition, decompression and removal of fish from the pressure chamber takes a considerable amount of time. This posed ethical concerns for the welfare of thefish.

### Experiment ^#^3: the normobaric heart rate response to increased temperature

Lumpfish (*n* = 12, 544.5 ± 19.1 g, 23.7 ± 0.4 cm) were implanted with loggers, four at a time, then returned to their holding tank for recovery for 72 hours. After recovery, the fish were transferred into individual buckets (26.5 cm in diameter × 23.5 cm deep, 8 l) in a water bath with flowing seawater and sufficient aeration to maintain water oxygen levels near 100% saturation. The lumpfish were given 24 hours at 12°C to acclimate to the buckets, during which time, photoperiod was maintained at 14-hour light:10-hourdark.

On the morning following transfer, the pre-programmed micro-HRT loggers began saving ECGs and recording *f*_H_ (100 Hz for 6 seconds) and temperature every 5 minutes at 8:00 AM. At 9:00 AM, water temperature was increased by 2°C h^−1^ to a maximum of 22°C (*n* = 8). Some lumpfish were also held at 12°C to serve as time-matched controls (*n* = 4).

Following all experiments, the fish were euthanized in 0.6 g l^−1^ MS-222 in order to perform post-mortem dissections and recover the data. Post-mortem dissections were conducted to record the distance from the front of the logger to the pericardium, the logger’s final position, to look for any signs of inflammation or infection and to determine sex based on the absence (i.e. immature fish) or presence of ovaries or testes.

### Calculation of heart rate parameters

All reported measurements of *f*_H_ were calculated manually using a previously described method (Zrini and Gamperl, 2021). Briefly, the average time between R wave peaks was measured (in seconds), and then 60 was divided by this number to obtain the fish’s *f*_H_ in beats per minute (bpm). Quality index (QI) measurements for each ECG also were provided by the Mercury software (with QI_0_ indicating very good quality, QI_1_ and QI_2_ indicating decreasing quality and QI_3_ meaning that no R-R interval was detected). The absolute difference between *f*_H_ values calculated by the on-board logger algorithm and those manually calculated ranged from 0 to 389 bpm (avg. absolute difference: QI_0_ = 1.4 bpm, QI_1_ = 9.1 bpm, QI_2_ = 15.9 bpm and QI_3_ = 21.9 bpm). When ECG artefacts made the PQRS complex unidentifiable, manual calculation was not possible and the data were not included. Percentage change in *f*_H_ was calculated for each fish based on initial *f*_H_ values in each experiment (e.g. as a percentage of 0 or 80 bar values prior to changes in temperature of oxygen level). Heart rate variability (HRV) was calculated as the standard deviation of time between successive R wave peaks (in ms). Heart rate scope (bpm) was determined in the pressure-exposed fish (Experiments ^#^1 and ^#^2) as the difference between mean *f*_H_ at 0 bar and that measured once 80 bar was reached.

### Lumpfish activity

Video was recorded during all experiments by connecting the pipe inspection camera in the view ports of the IPOCAMP’s lid to a laptop running VideoVelocity (CandyLabs, Vancouver, Canada). From these videos, the activity of all individuals was scored by assigning fish with a rank for each 10-minute period during exposure to pressure: 0 represented fish that were completely inactive; 1 represented fish that were mostly inactive but had some spontaneous activity; 2 represented fish that were mostly active, but had some periods of rest; and 3 represented fish that were active for the entire 10-minute period. Activity was ranked for all pressure steps in Experiments ^#^1 and ^#^2.

### Statistical analyses

To assess the effects of surgical recovery on *f*_H_, linear mixed-effects (LME) models were used with photoperiod (day time between 6:00 AM and 7:59 PM and night time between 8:00 PM and 5:59 AM) and day (where N1 was the first night following surgery and D1–D5 represent the subsequent days) as the fixed-effects, an interaction term, and fish as a random factor.

The effects of pressure on *f*_H_, the percentage change in *f*_H_, HRV, activity rank and the percentage of good quality (i.e. QI_0_) ECGs were assessed using LME models with pressure step (0, 20, 35, 50, 65 and 80 bar, and either the reverse during decompression or 10-minute increments when pressure was held at 80 bar) and treatment (control fish at 0 bar or pressure-exposed fish) as the fixed effects, an interaction term and fish as a random factor. For all pressure-exposed fish in Experiments ^#^1 and 2, the effects of sex (immature, female, male), temperature (10°C or 12°C) and activity rank at 0 bar on resting *f*_H_ (at 0 bar) or *f*_H_ scope (0 vs. 80 bar) were analysed by one-way ANOVAs and unpaired *t*-tests, while their relationship with weight (g), length (cm) and activity during pressure exposure was assessed using linear regressions. Lastly, the effect of resting *f*_H_ on *f*_H_ scope was determined using linear regression.

Linear regression analysis was also used to examine the relationships between *f*_H_ and the percentage change in *f*_H_ for each environmental parameter (decreased temperature, increased temperature, decreased oxygen and acute warming protocol on data up to 20.8°C), including determining whether the slopes and intercepts were different between treatments. Finally, an LME model with temperature/oxygen and treatment as fixed effects, an interaction term and fish as a random factor was used to assess changes in the percentage of QI_0_ECGs.

The LME models were performed in RStudio (v. 1.2.1335, RStudio Inc., Boston, MA, USA; http://www.rstudio.com) with the nlme package (Pinheiro *et al.*, 2020), while the other analyses were performed using Prism 7 (GraphPad Software, Inc., San Diego, CA, USA). Assumptions of normality, homogeneity and independence were analysed by visual inspection of Q-Q plots and histograms of the residuals, residual-fit plots and residual lag plots, respectively, for data analysed in RStudio. The estimated marginal means, or emmeans, package (Singmann *et al*., 2019) was used to perform Bonferroni post-hoc tests on all LME models and Tukey’s multiple comparison tests were performed following one-way ANOVAs. The level of statistical significance was *P* < 0.05. All values presented in the text are means ± standard errors of the mean (S.E.M.).

## Results

### Heart rate recovery and diel patterns post-surgery

**Figure 2:**
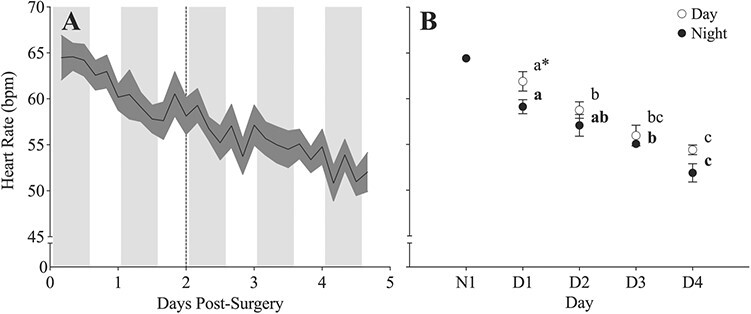
Moving average (**A**) and average (**B**) heart rate (*f*_H_, bpm) values in free-swimming lumpfish (*n* = 14) recorded every 4 hours for 5 days following the surgical implantation of the Star-Oddi micro-HRT logger. After 48 hours (dotted line), two lumpfish were transferred to containers inside the tank to be fasted for an additional ~66 hours. (A) Fish were on a 14-hour light:10-hour dark photoperiod (grey bars represent periods of darkness/night-time). (B) Open symbols represent day-time, while dark symbols represent night-time values. Dissimilar lower-case letters indicate a significant difference within a photoperiod group (for night-time values the letters are bolded), while an asterisk represents a significant difference between day-time and night-time values at each measurement point. The symbols represent means ± S.E.M. with each value representing the average of 3 data points per fish. Note: N1 was not included in the analysis.

Following implantation of the micro-HRT loggers, the *f*_H_ of lumpfish recovering in the holding tank at 10°C was recorded for 5 days (Fig. 2A). Average daily *f*_H_ decreased significantly during the recovery period (*P* < 0.0001; Fig. 2B; Table S1), from 61.8 ± 0.9 and 59.1 ± 1.1 bpm (day-time and night-time values) on the first day to 54.4 ± 0.9 and 51.9 ± 1.0 bpm on the final day of recovery. There was also a significant effect of photoperiod (*P* = 0.0015). However, the diel variation was relatively small (2–4 bpm) and only significant on the first day post-surgery.

### Experiment ^#^1: the heart rate response to hydrostatic pressure and the fish’s maximum post-exercise heart rate

**Figure 3:**
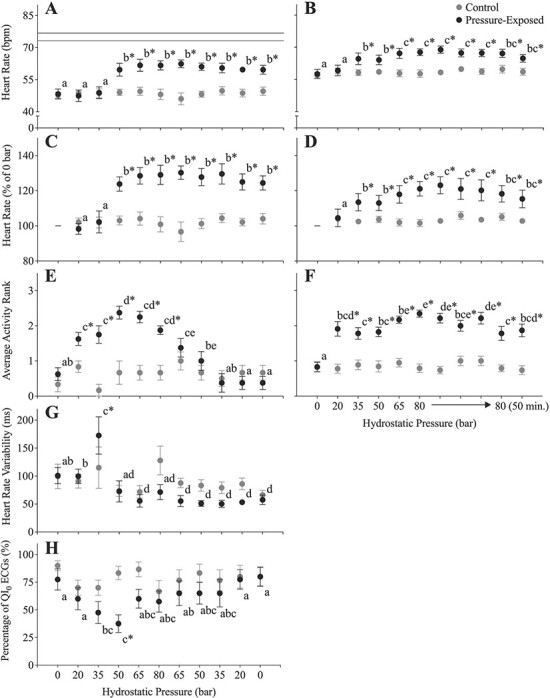
Lumpfish were held in the IPOCAMP at atmospheric pressure (0 bar, grey symbols) or exposed to hydrostatic pressure in a step-wise protocol (black symbols). Pressure was initially increased to 20 bar over 2 minutes, then held for 8 minutes. Pressure was then increased in a similar manner to 35, 50, 65 and 80 bar, followed by decompression in the opposite sequence (**A**, **C**, **E**, **G**, **H**; controls and 8 pressure exposed) or held at 80 bar for 1 hour (**B**, **D**, **F**; 19 controls and 23 pressure exposed). Heart rate (*f*_H_ in bpm; A and B), the percentage change in *f*_H_ (as a % of resting values at 0 bar; C and D) and HRV (in ms; G) were manually calculated from ECGs and the percentage of QI_0_ ECGs (H) were provided by the Star-Oddi Mercury software. The average activity rank (E and F) was determined from video recordings; where 0 represents fish that were completely inactive, 1 represents fish that were mostly inactive but had some spontaneous activity, 2 represents fish that were mostly active but had some periods of inactivity and 3 represents fish that were active for the entire 10-minute period. Maximum *f*_H_ post-chase following removal from the IPOCAMP is represented as a grey (control; *n* = 4) or line in panel A. Dissimilar lower-case letters indicate a significant difference within the pressure-exposed group (no differences existed in the control group), while an asterisk indicates a significant difference (*P* < 0.05) between the pressure-exposed and control groups at a particular pressure step. Data were recorded every 2 minutes and the symbols represent means ± S.E.M (5 per fish).

Heart rate, the percentage change in *f*_H_, activity, HRV and percentage of QI_0_ ECGs remained constant in the control fish (Fig. 3). At 10°C, hydrostatic pressure had a significant effect on *f*_H_ and the percentage change in *f*_H_ (*P* = 0.0025; *P* = 0.0012; Table S2). Heart rate began to increase between 35 and 50 bar, and while further increases were limited, *f*_H_ reached 61.5 ± 1.7 bpm (129.1 ± 3.8% of initial values) by 80 bar as compared to 48.1 ± 1.4 bpm in the control fish at the same time point (Fig. 3A). The lumpfish’s *f*_H_ remained elevated during decompression. Following removal from the IPOCAMP, maximum post-exercise *f*_H_ was 73.2 ± 1.4 and 76.8 ± 1.2 bpm (in control and pressure-exposed fish, respectively), suggesting that the pressure-induced increase in *f*_H_ was only ~47% of the fish’s scope for increases in *f*_H_ (Fig. 3A). Pressure significantly increased the activity of the fish exposed to 80 bar (*P* = 0.0006; Fig. 3E). At 0 bar, pressure-exposed fish had an average activity rank of 0.63 ± 0.18 and fish were either not moving (rank = 0) or swimming very little (rank = 1). Initial increases in activity began immediately upon compression, and activity rank peaked at 50 bar at 2.38 ± 0.18. Activity gradually decreased to normal levels during decompression.

**Figure 4:**
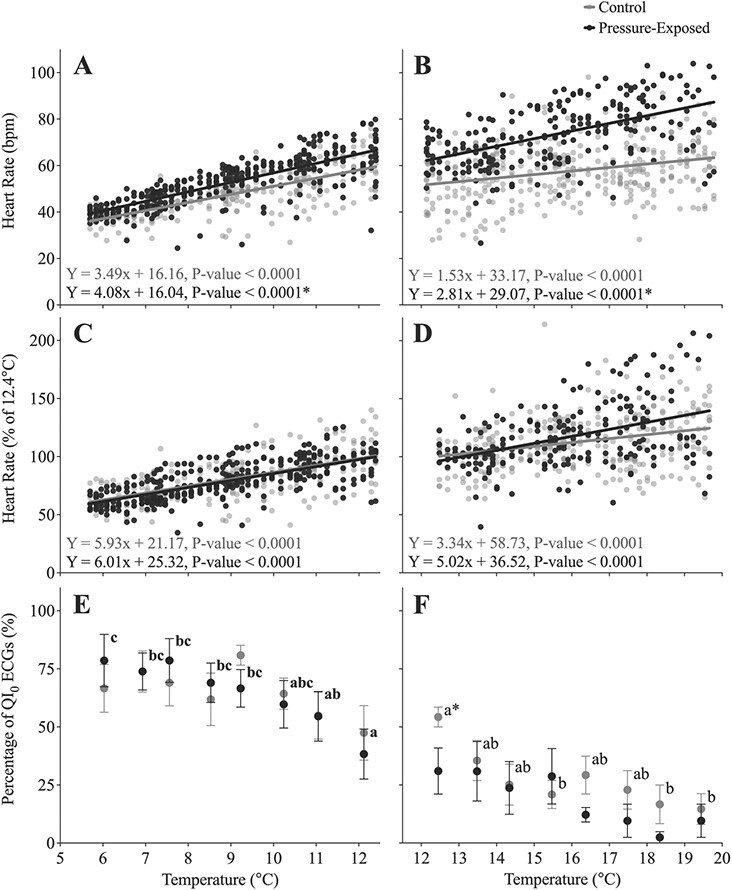
Lumpfish held at atmospheric pressure (grey symbols; 7 per experiment) or exposed to 80 bar of pressure (black symbols; 7 per experiment) were exposed to decreasing (at 2°C h^−1^; **A**, **C**, **E**) or increasing (at 2°C h^−1^; **B**, **D**, **F**) temperature in the IPOCAMP chamber. Heart rate (*f*_H_ in bpm; A and B) and the percentage change in *f*_H_ (as a % of resting values at 0 or 80 bar; C and D) were manually calculated from the ECGs. An asterisk indicates a significant difference between the slopes for relationships between control and pressure-exposed groups. The percentage of QI_0_ ECGs (E and F) was provided by the Star-Oddi Mercury software. In these panels, significant differences within the control (regular letters) or pressure-exposed groups (bold letters) are represented by dissimilar lower-case letters and an asterisk indicates a significant difference (*P* < 0.05) between the pressure-exposed and control groups at a particular temperature step. Data were recorded every 5 minutes, and the symbols represent means ± S.E.M (6 values per fish).

Overall, treatment did not significantly affect HRV or the quality of ECGs (*P* = 0.2932 and *P* = 0.0519, respectively; Table S2). However, there was a significant interaction between treatment and ‘pressure step’. In the pressure-exposed group, HRV decreased from 100.9 ± 14.5 ms at the beginning to 71.4 ± 13.5 ms at 80 bar and to 57.6 ± 8.5 ms after decompression (Fig. 3G). Interestingly, HRV spiked to ~173 ms at 35 bar, this value significantly higher than measured in the control group at this pressure. The percentage of QI_0_ ECGs fell from 0 to 50 bar, and then gradually returned to initial values; the values at 35 and 50 bar were significantly different from that of the control group at these pressures (Fig. 3H). Interestingly, 35–50 bar of pressure corresponded to the beginning of *f*_H_ increases, and the relationship between the percentage of QI_0_ values and pressure was a mirror image of that for activity.

### Experiment ^#^2: influence of hydrostatic pressure on the heart rate response to changes in temperature and hypoxia

At 12°C, the effects of hydrostatic pressure were similar to those at 10°C. However, *f*_H_ began to increase at 35 bar instead of 50 bar (Fig. 3B). Heart rate was significantly elevated by hydrostatic pressure (*P* = 0.0053) throughout the period of exposure, and this was also reflected in values of *f*_H_ when expressed as a percentage of initial values (*P* < 0.0001 for both parameters; Table S3). Heart rate did not change over the experiment in control fish. When pressure reached 80 bar, *f*_H_ was 67.7 ± 1.6 bpm compared to 57.7 ± 1.7 bpm in control fish at the same sampling point (i.e. 121.1 ± 4.1% of initial values) and *f*_H_ remained elevated above control values after 1 hour of acclimation to 80 bar. Similar to Experiment ^#^1, the lumpfish were significantly more active when exposed to increased pressure (*P* < 0.0001). Activity peaked at 2.35 ± 0.12 at 80 bar and remained elevated for the 50 minutes of exposure to 80 bar of pressure.

When data from fish in Experiments ^#^1 and ^#^2 were combined, weight and length had no effect on resting *f*_H_ (i.e. values at 0 bar), but sex significantly affected this parameter (*P* = 0.015), with females having a 14.6- and 14.1-bpm higher *f*_H_ than immature or male fish, respectively (*P* = 0.014 and *P* = 0.092; Fig. S2; Table S4). Neither weight, length, sex, temperature nor activity affected the *f*_H_ scope between 0 and 80 bar (*P* > 0.05; Fig. S2; Table S4). However, this *f*_H_ scope was negatively correlated with resting *f*_H_, with fish with high *f*_H_ values at 0 bar having a smaller increase in *f*_H_, or a decrease in *f*_H_, in response to pressure-exposure (*P* < 0.0001; Fig. S2; Table S4).

In control and pressure-exposed fish, *f*_H_ fell with decreasing temperature (*P* < 0.0001 and *P* < 0.0001, respectively; Fig. 4A; Table S5). The *f*_H_ of pressure-exposed fish was 7.0 bpm higher than that of control fish before the temperature began to decrease (i.e. at 80 bar), and the slopes of the relationship between *f*_H_ and temperature were significantly different (*P* = 0.007; Table S5) as *f*_H_ in both groups was 38–39 bpm at ~5.7°C. In contrast, the Q_10_ values, and the slopes of the relationships between relative *f*_H_ (as a percentage of the initial value) and temperature, were not significantly different between the control and pressure-exposed groups (Fig. 4A; Table 1).

**Table 1:** Summary of Q_10_ and average maximum values of *f*_H_ recorded in lumpfish

Experiment	Location	Treatment	Maximum *f*_H_ (bpm)	Temperature (°C)	Q_10_
^#^1	Bucket	Chase – Control	73	10	-
		Chase – Pressure	77	10	-
^#^2	IPOCAMP	Control	-	12.2–6.1	2.10
		Pressure	-	12.0–5.9	2.07
^#^2	IPOCAMP	Control	64	12.4–19.3	1.37
		Pressure	83	12.5–19.6	1.39
^#^3	Bucket	Acute Warming	81	12.3–20.8	1.67

**Figure 5:**
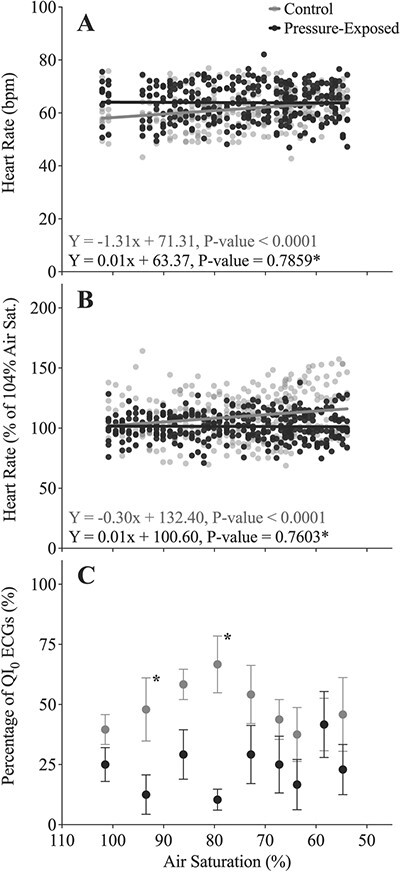
(**A**) Heart rate (*f*_H_, bpm) and (**B**) the percentage change in *f*_H_ (as a % of initial values at 0 or 80 bar) in lumpfish exposed to decreasing oxygen levels (air saturation, %) in the IPOCAMP chamber over 3–4 hours. Prior to the decrease in oxygen levels, lumpfish were held at atmospheric pressure (black circles; *n* = 8) or exposed to 80 bar of pressure (grey circles; *n* = 8). An asterisk represents a significant difference in the slopes of the relationships between control and pressure-exposed lumpfish. (**C**) The percentage of QI_0_ ECGs were provided by the Star-Oddi Mercury software, and in this panel, an asterisk represents a significant difference between pressure-exposed and control groups at a particular oxygen level. Within each treatment group, there were no differences in the quality of ECGs. Data were recorded every 5 minutes, and the symbols represent means ± S.E.M (6 values per fish).

Heart rate was 16.0 bpm higher in the pressure-exposed group before temperature was raised and increased in both control and pressure-exposed fish with temperature (*P* < 0.0001 and *P* < 0.0001, respectively; Fig. 4B). The slope of this relationship was significantly greater in the pressure-exposed group (*P* = 0.0078; Table S5). However, the Q_10_ values for *f*_H_ (1.37 and 1.39) and the slopes of the relationships between temperature and relative *f*_H_ (i.e. as a percentage of initial values) were again not significantly different (*P* = 0.1069). When the two temperature challenges are considered together, the *f*_H_ of control and pressure-exposed lumpfish increased from 38 to 64 bpm and 39 to 83 bpm, respectively, from 5 to 20°C.

When held at atmospheric pressure in the IPOCAMP, the *f*_H_ of lumpfish increased slightly with decreasing oxygen (*P* < 0.0001; Fig. 5A; Table S5), i.e. from 58.7 ± 3.2 bpm at 106% air sat. to 62.3 ± 2.9 bpm at 57% air sat. However, exposure to 80 bar of hydrostatic pressure eliminated the effect of decreasing oxygen level, i.e. *f*_H_ remained unchanged from 103% to 57% air sat. (*P* = 0.7859). The same relationships were evident when *f*_H_ data was calculated as a percentage of initial values (Fig. 5B; Table S5).

Hydrostatic pressure had a significant effect on the percentage of QI_0_ ECGs during the decreasing oxygen experiment (*P* = 0.0292; Table S6), but not in the decreasing or increasing temperature experiments (*P* = 0.9784 and *P* = 0.1939, respectively). On average, QI_0_ ECG values were ~25% fewer in pressure-exposed fish compared to control fish in the hypoxia experiment (48.4% vs. 23.6%). Conversely, decreasing or increasing temperature, but not decreasing oxygen (*P* < 0.0001, *P* < 0.0001 and *P* = 0.2698, respectively; Fig. 4E and F; Fig. 5C) strongly affected the quality of the ECGs. Overall, the percentage of QI_0_ ECGs fell from 72.6% at 6.0°C to 12.3% at 19.4°C.

### Experiment ^#^3: the normobaric heart rate response to increased temperature

**Figure 6:**
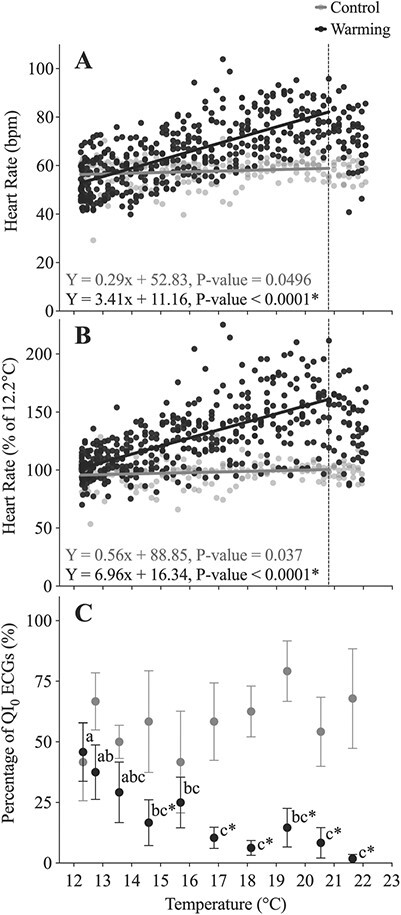
(**A**) Heart rate (*f*_H_, bpm) and (**B**) the percentage change in *f*_H_ (as a % of initial values at 12.2°C) in lumpfish during an acute warming protocol (black symbols; *n* = 12) in a water table, where temperature was increased at 2°C h^−1^ vs. when fish were held at a constant temperature of 12°C (grey symbols; *n* = 4). An asterisk indicates a significant difference (*P* < 0.05) in the slopes between control fish and those exposed to increasing temperature. (**C**) The percentage of QI_0_ ECGs was provided by the Star-Oddi Mercury software. In this panel, significant differences within the warming group (there were no differences in the control group) are represented by dissimilar lower-case letters, and an asterisk represents a difference between the warming and control group at a particular temperature. Data were recorded every 5 minutes, and the symbols represent means ± S.E.M (6 per fish). The dotted line indicates the temperature of 20.8°C. Beyond this temperature the *f*_H_ of the lumpfish began to decrease, and thus, these data were not included in the linear regression.

When lumpfish underwent an acute warming protocol under normobaric conditions, *f*_H_ was 52.4 ± 2.5 bpm at 12.3°C, peaked at 81.0 ± 3.6 bpm at 20.8°C (*P* < 0.0001) and then declined to 71.7 ± 3.6 bpm by 22.1°C (a scope of ~29 bpm between 12.3°C and 20.8°C; Q_10_ = 1.67) (Fig. 6A; Table 1; Table S5). This value for maximum *f*_H_ was ~17 bpm higher than the corresponding value reached in fish held at atmospheric pressure in the IPOCAMP (~64 bpm at 19.6°C), but comparable to the maximum *f*_H_ recorded for lumpfish at 80 bar (~83 bpm at 19.6°C). Increasing temperature also resulted in a significant decrease in the percentage of QI_0_ ECGs; this value being significantly lower than the time-matched control group at temperatures >15°C (*P* = 0.0041; Fig. 6C; Table S6). Overall, the percentage of QI_0_ values decreased from 45.8% at 12.3°C to 1.8% at 21.6°C (compared to 41.7% and 67.9% in the time-matched controls).

## Discussion

### Post-surgical recovery and diel patterns in heart rate

While not a primary goal of this research, the *f*_H_ of lumpfish was recorded following the implantation of micro-HRT loggers to monitor recovery. This is because netting, handling, anaesthesia and surgery induce a physiological stress response in fish leading to increased *f*_H_ (Altimiras and Larsen, 2000; Hill and Forster, 2004; Rothwell *et al*., 2005; Grӓns *et al*., 2014). Initially, the day-time *f*_H_ of lumpfish was 62 bpm, but this value was ~60 bpm after 48 hours of recovery and 54 bpm by 5 days (at 10°C). Given the limited maximum *f*_H_ in this species (~80 bpm, see below), it might be expected that they would have a low resting *f*_H_ so that they would have a scope for *f*_H_ comparable to other teleost species (1.8- to 2.6-fold increase in *f*_H_ during CT_MAX_ tests; Gollock *et al*., 2006; Clark *et al*., 2008; Vornanen *et al*., 2014; Penney *et al*., 2014; Motyka *et al*., 2017). These data may indicate that the lumpfish had not fully recovered from surgery and that this explains their high resting *f*_H_. This would agree with recent research on Atlantic salmon showing that while *f*_H_ decreases most during the first week post-implantation it continues to fall for 2–3 weeks post-surgery (Hvas *et al.*, 2020; Zrini and Gamperl, 2021). However, it is also possible given the low aerobic scope and U_crit_ of this species (Hvas *et al*., 2018) that a large scope for *f*_H_ is not required.

Overall, there was a significant effect of photoperiod on the *f*_H_ of lumpfish during the recovery period (Fig. 2). However, day-time values were only significantly different than night-time values on the first day post-surgery, and diel variations in *f*_H_ were relatively small (2 to 4 bpm). Conversely, Atlantic salmon had average diel variations of 7 bpm (and up to 14 bpm) at similar temperatures and time points post-surgery that were synchronous with changes in swimming activity (Zrini and Gamperl, 2021). It is possible that the dampened diel variations in *f*_H_ were related to low day-time activity of the lumpfish; however, this parameter was not monitored in this study. While limited evidence suggests that lumpfish behave diurnally (Imsland *et al.*, 2015; Leclercq *et al*., 2018), further investigation is needed to understand diel patterns of behaviour and physiology in this species, especially given the flexibility of these characteristics.

### Heart rate response to hydrostatic pressure

The primary goal of this research was to investigate the effect of hydrostatic pressure on the *f*_H_ of lumpfish. In response to an acute exposure to 80 bar of pressure, the *f*_H_ of 10°C-acclimated lumpfish increased by ~14 bpm (20–30%) above resting values (Fig. 3A and C). Further, this tachycardia was sustained during 1 hour at 80 bar (Fig. 3B and D) and only diminished slightly during decompression. Previous research on this topic is extremely limited, possibly due to technical limitations (Guerrero *et al*., 2000). However, our results are generally consistent with other studies that have measured the effect of hydrostatic pressure on *f*_H_ in fishes at temperatures within the middle of a species’ thermal range. For example, Sébert and Barthélémy (1985b) reported that (i) exposure to ~101 bar of pressure increased the *f*_H_ of freshwater eels acclimated to 15–20°C by ~30–80% and that tachycardia was sustained during the 1 hour of pressure exposure; and (ii) while *f*_H_ did fall to some degree during decompression, it was still not back to pre-exposure levels by 1 hour. Also, Naroska (1968) showed that abrupt compression to ~50 bar produced a transient tachycardia in 5°C eel pout (*Zoarces viviparous*) and Belaud *et al*. (1976) found that pressure induced a tachycardia below the temperature of 24.5°C in eels (c.f. Sébert and Macdonald, 1993). Further, our results agree with studies on the effects of pressure on oxygen consumption (ṀO_2_) in a variety of teleost species (reviewed in Sebert and Macdonald, 1993). While the relationship between ṀO_2_ and *f*_H_ can be influenced by changes in stroke volume (Farrell and Smith, 2017), this latter data is still highly relevant to our conclusions (Armstrong, 1986; Lucas, 1994); especially given the limited *f*_H_ data on the following topics.

With regards to the pressure at which increases in *f*_H_ begin in fishes, the data are difficult to compare as the maximum hydrostatic pressure the fish is exposed, the rate of compression and temperature all influence the *f*_H_ response to increased pressure (Sébert and Macdonald, 1993). In this study, *f*_H_ began to increase between 30 and 50 bar and this is consistent with Sébert and Barthélémy (1985b) where, after no change or a brief bradycardia in some eels, *f*_H_ began to increase at 40 to 50 bar. In contrast, the pressure at which the ṀO_2_ of male and female eels began to increase was between 50 and 80 bar (Scaion *et al*., 2008a), and Speers-Roesch *et al*. (2004) demonstrated that pressures as low as 3 bar increased ṀO_2_ in the bloater (*Coregonus hoyi*). The latter data suggest that the normal depth range of a given species likely has a significant effect on the sensitivity of their responses to increasing pressure. In this study, the increase in *f*_H_ induced by hydrostatic pressure was only ~47% of the available scope for *f*_H_. Again, this is consistent with Sébert and Barthélémy (1985b) who reported that while the maximum temperature-induced *f*_H_ in eels is ~120 bpm, *f*_H_ when exposed to ~101 bar did not exceed 60 bpm. These data indicate that fish at high pressure (at least those whose life history includes excursions to the applied pressures) still have a considerable scope available for increases in *f*_H_.

Many authors attribute the reported increases in ṀO_2_ to a simultaneous increase in motor activity during compression (Sébert and Barthélémy, 1985a; Simon *et al*., 1989; Sébert *et al*., 1997; Vettier *et al*., 2003), which Speers-Roesch *et al*. (2004) suggested was partially related to compression of the swim bladder in bloater, and thus a loss of buoyancy. Lumpfish do not possess a swim bladder (Powell *et al*., 2017) and were supported by a platform in the pressure chamber. Nonetheless, they became agitated and more active during compression and this hyperactivity was maintained for 1 hour at 80 bar (see Fig. 3E and F); a behavioural response that may simply be an attempt to escape this novel situation. Sébert and Barthélémy (1985b) found that increases in motor activity during compression in eels were associated with tachycardia. These data strongly suggest that increased activity was primarily responsible for the increase in *f*_H_ with pressure exposure. However, it is also possible that the observed tachycardia was in part related to alterations in the neurohormonal control of *f*_H_. This conclusion is based on three lines of evidence. First, exposing isolated eel hearts (which are free of neurohormonal control) to increased pressure resulted in a pronounced bradycardia, not tachycardia (Pennec *et al*., 1988). Second, Belaud *et al*. (1976) and Sébert and Barthélémy (1985b) show that atropine, and adrenergic agonists and antagonists, markedly alter the magnitude of the tachycardic response when eels are exposed to increased hydrostatic pressure. Third, HRV was considerably lower in the pressure-exposed group near the end of the compression period at 80 bar and remained lower during decompression (Fig. 3G). The mechanisms involved in pressure-induced increases in *f*_H_ require investigation but could be related to alterations in cholinergic or adrenergic tone, or receptor function/affinity associated with changes in pacemaker cell membrane fluidity (Sébert and Barthélémy, 1985b). Further, several studies have provided evidence that a pressure-induced decrease in membrane fluidity, or ‘rigidification’, results in ‘compression-induced histotoxic hypoxia’ in fish (Sébert and Barthélémy, 1985a; Sébert *et al*., 1987; Sébert *et al*., 1993). However, this latter hypothesis/phenomenon is controversial and is not supported by data on the ṀO_2_ of permeabilized red muscle fibres (Scaion *et al*., 2008b) or blood PO_2_ (Sébert *et al*., 1997).

By combining the data from all of the pressure-exposed experiments, it was possible to also investigate the influence of acclimation temperature, sex, mass, length and activity on the initial *f*_H_ and scope for *f*_H_ in response to compression (Fig. S2). Despite females having a higher resting *f*_H_, we report that sex, weight, length and activity did not affect the scope for *f*_H_ during compression. The lack of an effect of sex on pressure-induced physiology in lumpfish at 10–12°C is consistent with the ṀO_2_ data for eels at temperatures below 15°C (Scaion *et al*., 2008a). However, these authors also report that the ṀO_2_ of female eels was much more sensitive at 22°C as compared to males, and thus the effects of sex on hydrostatic pressure-related changes in the *f*_H_ of fishes cannot be excluded. Interestingly, the initial *f*_H_ of lumpfish at 0 bar significantly affected the *f*_H_ scope in response to the stress of pressure exposure (Fig. S2). Previous research compliments these results, as the capacity for rainbow trout to increase *f*_H_ in response to exercise was highly dependent on their resting *f*_H_ (Brijs *et al*., 2019). It is possible that variation in the intrinsic *f*_H_ of individuals, or in stress caused by the transfer to the IPOCAMP, resulted in a high allostatic load for some fish and that this reduced their ability to increase *f*_H_ in response to pressure.

### Influence of hydrostatic pressure on the heart rate response to changes in temperature and hypoxia

After lumpfish were exposed to 80 bar of pressure for 1 hour, changing temperature resulted in a linear change in *f*_H_. While the relationship was steeper for absolute *f*_H_ in fish exposed to hydrostatic pressure, the relationship was similar to control fish when the elevated *f*_H_ in pressure exposed fish at 12°C was taken into account (Fig. 4). These results suggest that while hydrostatic pressure does have an effect on resting *f*_H_, it does not influence the sensitivity of *f*_H_ to changes in temperature. This finding was quite surprising as Scaion *et al*. (2008a) showed that temperature had a significant effect on the sensitivity of ṀO_2_ to increases in hydrostatic pressure, and Sébert *et al*. (1995b) reported that exposing eels to a 5°C temperature increase (from 15°C to 20°C) concomitantly with an increase in pressure to ~101 bar reduced the acute increase in ṀO_2_ by ~50%. Finally, while tachycardia is seen in pressure-exposed eels at lower temperatures, this response changes to a bradycardia at temperatures near this species’ critical thermal maximum (CT_MAX_; ~ 31°C) (Belaud *et al*., 1976; Claësson *et al*., 2016). The disparity in response to temperature between our study and these studies may be related to species or methodological differences. Most importantly, we exposed the lumpfish to elevated pressure for 1 hour prior to any changes in temperature, whereas the eels were exposed to changes in temperature either before, or in concert with, changes in hydrostatic pressure.

In the lumpfish, decreasing water PO_2_ at atmospheric pressure from ~100% to ~55% saturation resulted in a slight increase in *f*_H_ (by ~5 bpm). This minor increase in *f*_H_ was somewhat surprising as *f*_H_ generally does not change as water PO_2_ is lowered to the point of bradycardia. However, such a response has been seen in several other fish species including the Atlantic cod (*Gadus morhua*) (Gamperl and Driedzic, 2009; Petersen and Gamperl, 2011). Exposure to pressure eliminated the small increase in *f*_H_ that was observed in the control fish (Fig. 5). This is an interesting observation, and while the mechanism(s) mediating this difference is/are unknown, these data suggest that fish experiencing increased hydrostatic pressure and moderate hypoxia may have a very limited scope for increases in *f*_H_. Bradycardia is typically recorded at oxygen levels similar or slightly higher than a species’ critical oxygen tension, P_crit_ (e.g. see Marvin and Heath, 1968; Gehrke *et al*., 1988; Speers-Roesch *et al*., 2010). Therefore, it is very likely that bradycardia was not recorded in this study because the lumpfish did not reach their P_crit_ (~40% air saturation at 12°C; Ern *et al*., 2016). Future experiments are being planned to examine if hydrostatic pressure affects the oxygen level at which bradycardia is initiated and the magnitude of the decrease in *f*_H_.

Ultimately, the most relevant experimental scenario would be one that accurately reflects the environmental and behavioural challenges of a vertical migration, i.e. simultaneous increases in pressure and decreases in temperature and water oxygen levels while the fish is actively swimming (e.g. fish dealing with changing water currents or evading predation).

### Maximum exercise and temperature-induced heart rate of lumpfish

Given the low maximum *f*_H_ recorded in the IPOCAMP at 20°C (63 bpm) and following exercise at 10°C (73–77 bpm), an acute warming protocol was performed under normobaric conditions. The *f*_H_ of lumpfish increased up to 20.8°C (Q_10_ = 1.67; Table 1) and began falling as temperatures approached the lumpfish’s CT_MAX_ of 22°C (Fig. 6; Ern *et al*., 2016). This response is typical of that seen in other fish species, where *f*_H_ increases (at Q_10_ values ranging from 1.5 to 2.5) up until ~2°C before the fish’s CT_MAX_ (Gollock *et al*., 2006; Steinhausen *et al*., 2008; Gilbert *et al*., 2019). The highest individual *f*_H_ recorded in lumpfish was 95 bpm, while the highest average *f*_H_ at 20°C was 81 bpm. Thus, it appears that lumpfish have a low maximum *f*_H_ relative to fish species such as the channel catfish *Ictalurus punctatus* (150 bpm; Burleson and Silva, 2011) and salmonids (105–132 bpm; Clark *et al*., 2008; Steinhausen *et al*., 2008; Vornanen *et al*., 2014; Motyka *et al*., 2017) and more typical of those recorded in species such as the Atlantic cod (72 bpm; Gollock *et al*., 2006), winter flounder (*Pseudopleuronectes americanus*; 73 bpm; Mendonça and Gamperl, 2010) and European perch (*Perca fluviatilis*; 83 bpm; Jensen *et al*., 2017).

These results, combined with previous data, suggest that lumpfish are well adapted to a passive, yet still pelagic, lifestyle. Hvas *et al*. (2018) reported that lumpfish have a low critical swimming speed and aerobic scope due to a limited maximum ṀO_2_. The results of this study agree with the findings of Hvas *et al*. (2018), as lumpfish were found to have a low scope for *f*_H_ and a low maximum *f*_H_. Additionally, research shows that lumpfish have relatively low values of exercise-induced cortisol, glucose and lactate, which indicates that lumpfish have a limited capacity to perform exhaustive exercise (Clow *et al*., 2017; Jørgensen *et al*., 2017; Hvas *et al*., 2018). These physiological features are in contrast to most pelagic fish, which are built for strong swimming and aerobic performance, however, not surprising given the lumpfish’s globiform shape, weak tail musculature and uniquely docile nature (Hvas *et al*., 2018).

### Considerations when using data loggers in lumpfish

Temperature had a strong effect on the quality of ECGs recorded by the micro-HRT logger. While most *f*_H_ values could still be calculated by manually examining the ECG recordings, this is a concern for research being conducted at high temperatures or close to the CT_MAX_ of the species being studied. It has been suggested that low-quality ECGs are related to increased activity at higher temperatures because the potentials from aerobic muscles overlap with the ECG (Altimiras and Larsen, 2000). Interestingly, the percentage of QI_0_ ECGs also transiently decreased during compression, which was also associated with an increase in activity (Fig. 2). However, we do not believe that this was the main factor affecting ECG quality because the percentage of QI_0_ recordings in Atlantic salmon during a U_crit_ swim test never fell below 50% (Zrini and Gamperl, 2021). Instead, we believe that it was the low amplitude of the signal received by the logger that was the primary issue. In salmon, the R wave amplitude was ~510 mV (see Zrini and Gamperl, 2021), but it was only ~170 mV in the lumpfish (see Fig. S3). This low signal amplitude was not due to the size of the heart as the relative ventricular mass of lumpfish reared at 9°C is 0.94 (Hvas *et al*., 2018), and within the range of that reported for Atlantic salmon (Deitch *et al*., 2006; Anttila *et al*., 2015). Further, it is not that the lumpfish has a particularly large liver (e.g. the hepatosomatic index is only 2.5%; Hvas *et al*., 2018). However, the heart is relatively deep within the body cavity in lumpfish, and the liver’s position is such that it lies directly between the position of the logger and the heart. This may diminish the strength of the signal received by the data logger. It is possible that modifications may be able to be made to the logger’s design, or to the software/algorithms used to calculate *f*_H_, to enhance the logger’s usefulness for this species.

## Conclusions

The effects of hydrostatic pressure on the cardiovascular system of fish are poorly understood, and this is often attributed to the difficulty of obtaining physiological data while fish are at pressure (Guerrero *et al*., 2000; Shillito *et al*., 2014). With the miniaturization and growing popularity of biologgers for use in fish (Wilson *et al*., 2015), we are learning about the vertical movement patterns of marine species, but this also leads to further questions such as the following: how physiological perturbations associated with pressure influence their capacity to deal with other environmental challenges or how simultaneous changes in conditions such as temperature and oxygen levels affect the heart’s response to pressure. Star-Oddi micro-HRT loggers and IPOCAMP chambers were successfully used in this research to show that acute exposure to hydrostatic pressure produced a tachycardia in lumpfish, but that this had no effect on the slope of the temperature-*f*_H_ relationship when this pressure-induced increase was taken into account. In contrast, the minor increase in the *f*_H_ of control fish to decreasing water PO_2_ was eliminated by exposure to hydrostatic pressure. Lastly, lumpfish were found to have a low maximum *f*_H_ in response to exercise or a temperature increase close to their CT_MAX_ relative to other fishes. Our research suggests that pressure can influence the *f*_H_ response to environmental challenges and provides the first evidence that lumpfish have a limited capacity to increase *f*_H_.

## Funding

This work was supported by a Natural Sciences and Engineering Research Council of Canada Discovery Grant [#2016-0448 to A.K.G.] and partially supported by a Memorial University School of Graduate Studies Fellowship [to Z.A.Z.].

## Supplementary Material

Supplementary_Material_coab058
