# Supplementary material for: Does hydrostatic pressure influence lumpfish (Cyclopterus lumpus) heart rate and its response to environmental challenges?
Source: Conserv Physiol. 2021 Jul 22;9(1):coab058. doi: 10.1093/conphys/coab058 (PMC8299717; doi:10.1093/conphys/coab058)
Supplement: Supplementary_Material_coab058 [file supplementary_material_coab058.docx]

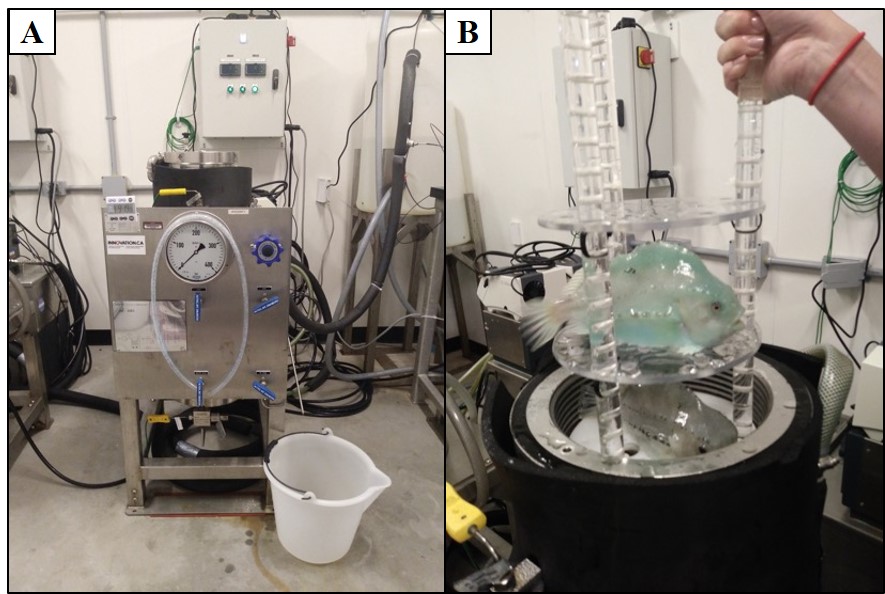


**Figure S1:** A) The IPOCAMP pressure chamber (19 L vessel, 60 cm high by 20 cm in diameter) in the Cold-Ocean and Deep-Sea Research Facility at the Ocean Science Centre. B) Lumpfish implanted with micro-HRT tags were placed, two at a time, onto platforms before being lowered into the IPOCAMP. The fish were acclimated to the chamber overnight at 0 bar of pressure (i.e., equivalent to atmospheric pressure at sea level).


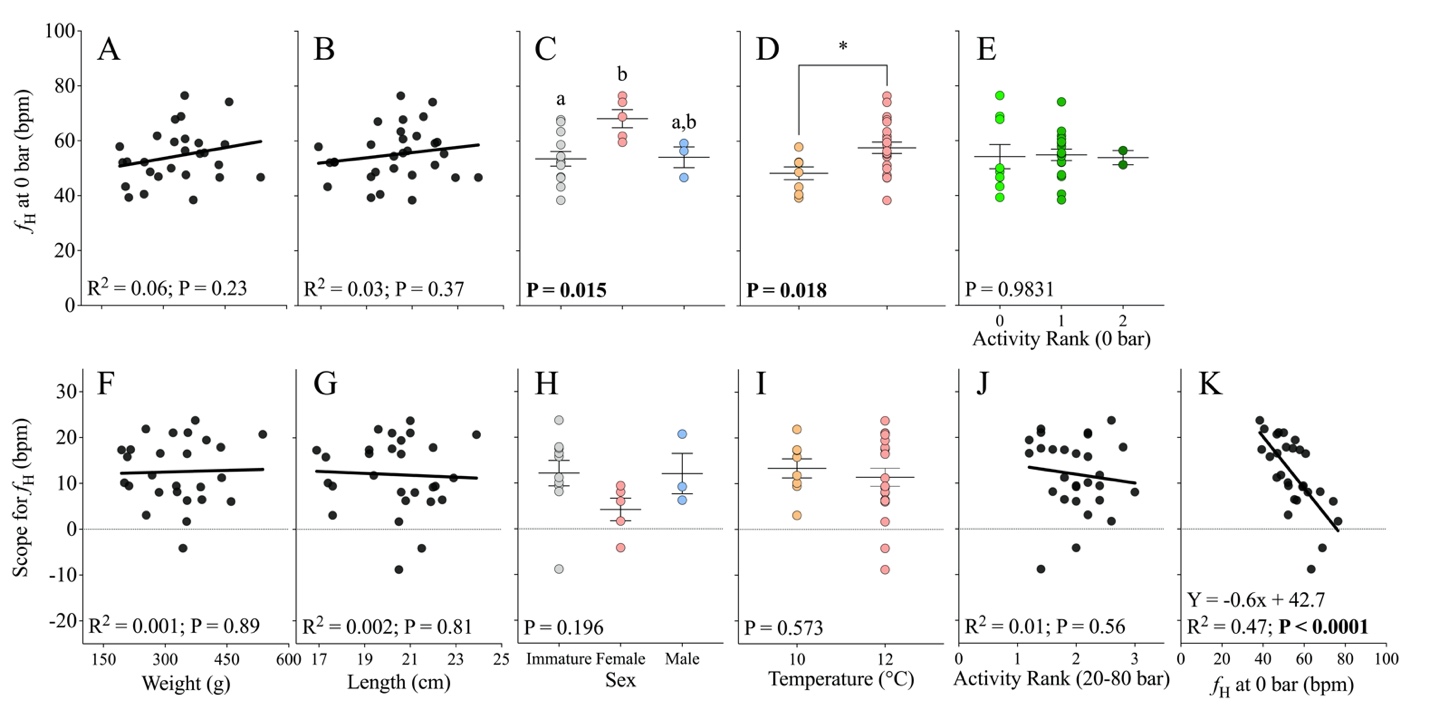


**Figure S2:** Lumpfish were exposed to hydrostatic pressure in a step-wise protocol in Exp ^#^1 (n=8) and ^#^2 (n=23), and the data was used to investigate the effects of weight (g; A and F), length (cm; B and *G*), sex (C and H), acclimation temperature (°C; D and I), and activity (E and J) on their initial *f*_H_ at 0 bar and their scope for *f*_H_ during compression between 0 and 80 bar. Additionally, the effect of initial *f*_H_ on the scope for *f*_H_ was also analyzed. Linear regressions, unpaired t-tests, ANOVAs and Tukey’s post-hoc tests performed in Prism were used to examine the effects of these factors. Significant P values (<0.05) are bolded. A difference in initial *f*_H_ at 0 bar is represented by differing lower case letters between sexes, and an asterisk between acclimation temperatures.


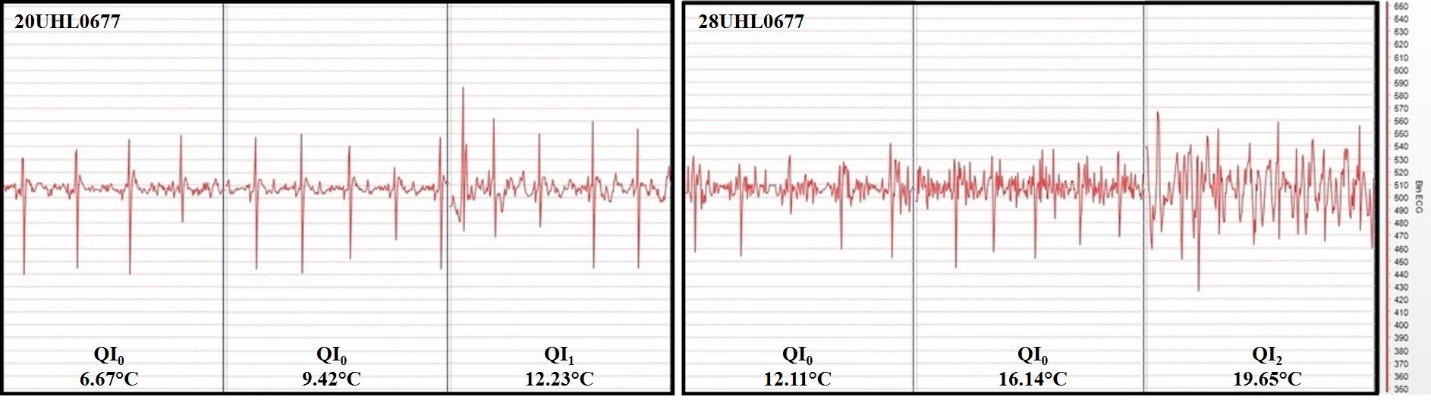


**Figure S3:** Electrocardiograms recorded in lumpfish exposed to decreasing (at 2°C h^-1^; left panel) or increasing (at 2°C h^-1^; right panel) temperature in the IPOCAMP chamber at atmospheric pressure (0 bar). These fish were chosen as their ECG recordings, and their response to temperature, were typical of what was observed for this species. Bin ECG represents the amplitude of the PQRS waveform and ranges from 0 to 1000 mV, but only the range from 350 to 650 mV is presented. The quality index (QI) was assigned to ECG recordings (QI_0_ means very good quality, QI_1_ and QI_2_ indicate decreasing quality and QI_3_ means no R-R interval was detected) by the Star-Oddi Mercury software.

**Table S1:** Summary of the LME model statistical outputs during post-surgical recovery.

| Independent Factor | Dependent Factor | NumDF | DenDF | F-value | P |
| --- | --- | --- | --- | --- | --- |
| *f*_H_ | (Intercept) | 1 | 285 | 2851.6 | **<0.0001** |
|  | Photoperiod Order | 3 | 285 | 24.5 | **<0.0001** |
|  | Photoperiod | 1 | 285 | 10.3 | **0.0015** |
|  | Interaction | 3 | 285 | 0.5 | 0.6336 |

LME models examined the effects of photoperiod (day-time / night-time), photoperiod order (days post-implantation), and their interaction on the heart rate of lumpfish over 5 days post-surgical recovery. Significant P values (<0.05) are represented in bold type. NumDF = degrees of freedom of the numerator of the F distribution ratio; DenDF = degrees of freedom of the denominator of the F distribution ratio.

**Table S2:** Summary of the LME model statistical outputs during pressure exposure (Exp. ^#^1).

| Independent Factor | Dependent Factor | NumDF | DenDF | F | P |
| --- | --- | --- | --- | --- | --- |
| *f*_H_ | (Intercept) | 1 | 683 | 2007.2 | **<0.0001** |
|  | Treatment | 1 | 12 | 14.4 | **0.0025** |
|  | Pressure Step | 10 | 683 | 23.9 | **<0.0001** |
|  | Interaction | 10 | 683 | 19.05 | **<0.0001** |
| % Change in *f*_H_ | (Intercept) | 1 | 617 | 2209.5 | **<0.0001** |
|  | Treatment | 1 | 12 | 17.8 | **0.0012** |
|  | Pressure Step | 9 | 617 | 15.7 | **<0.0001** |
|  | Interaction | 9 | 617 | 14.4 | **<0.0001** |
| Activity Rank | (Intercept) | 1 | 120 | 203.2 | **<0.0001** |
|  | Treatment | 1 | 12 | 21.4 | **0.0006** |
|  | Pressure Step | 10 | 120 | 9.7 | **<0.0001** |
|  | Interaction | 10 | 120 | 6.5 | **<0.0001** |
| HRV | (Intercept) | 1 | 679 | 291.1 | **<0.0001** |
|  | Treatment | 1 | 12 | 1.2 | 0.2932 |
|  | Pressure Step | 10 | 679 | 5.1 | **<0.0001** |
|  | Interaction | 10 | 679 | 3.7 | **<0.0001** |
| % of QI_0_ ECGs | (Intercept) | 1 | 119 | 409.6 | **<0.0001** |
|  | Treatment | 1 | 12 | 4.7 | 0.0519 |
|  | Pressure Step | 10 | 119 | 2.5 | **0.0104** |
|  | Interaction | 10 | 119 | 1.3 | 0.2458 |

LME models examined the effects of treatment (control vs. pressure-exposed), pressure (0, 20, 35, 50, 65, 80 bar and decompression in the opposite sequence) and their interaction, on *f*_H_, the percentage change in *f*_H_ (% of initial 0 bar values), heart rate variability (HRV), activity rank and the percentage of *f*_H_ values that were of ‘good’ quality (i.e., QI_0_). Significant P values (<0.05) are represented in bold type. *f*_H_ = heart rate; HRV = heart rate variability; NumDF = degrees of freedom of the numerator of the F distribution ratio; DenDF = degrees of freedom of the denominator of the F distribution ratio.

**Table S3:** Summary of the LME model statistical outputs during pressure exposure (Exp. ^#^2).

| Independent Factor | Dependent Factor | NumDF | DenDF | | F | P |
| --- | --- | --- | --- | --- | --- | --- |
| *f*_H_ | (Intercept) | 1 | | 1923 | 3900.9 | **<0.0001** |
|  | Treatment | 1 | | 43 | 8.5 | **0.0055** |
|  | Pressure Step | 10 | | 1923 | 10.1 | **<0.0001** |
|  | Interaction | 10 | | 1923 | 8.9 | **<0.0001** |
| % Change in *f*_H_ | (Intercept) | 1 | | 1719 | 2956.5 | **<0.0001** |
|  | Treatment | 1 | | 43 | 8.6 | **0.0053** |
|  | Pressure Step | 9 | | 1719 | 4.4 | **<0.0001** |
|  | Interaction | 9 | | 1719 | 6.8 | **<0.0001** |
| Activity Rank | (Intercept) | 1 | | 397 | 495.5 | **<0.0001** |
|  | Treatment | 1 | | 40 | 66.5 | **<0.0001** |
|  | Pressure Step | 10 | | 397 | 7.5 | **<0.0001** |
|  | Interaction | 10 | | 397 | 5.6 | **<0.0001** |

LME models examined the effects of treatment (control vs. pressure-exposed), pressure (20, 35, 50, 65, 80 bar and time at 80 bar), and their interaction, on *f*_H_, the percentage change in *f*_H_ [i.e., % of initial (0 bar) values] and activity rank. Significant P values (<0.05) are represented in bold type. *f*_H_ = heart rate; NumDF = degrees of freedom of the numerator of the F distribution ratio; DenDF = degrees of freedom of the denominator of the F distribution ratio.

**Table S4:** Summary of statistical outputs from pressure-exposed fish (Exp. ^#^1 and 2).

|  |  | F | R^2^ | P | N1 | N2 |
| --- | --- | --- | --- | --- | --- | --- |
| ANOVA and Tukey’s Post Hoc Summary | |  |  |  |  |  |
| *f*_H_ at 0 bar vs. Sex |  | 5.38 | 0.39 | **0.015** |  |  |
|  | I vs. F |  |  | **0.014** | 12 | 5 |
|  | I vs. M |  |  | 0.994 | 12 | 3 |
|  | F vs. M |  |  | 0.092 | 5 | 3 |
| *f*_H_ Scope vs. Sex |  | 1.81 | 0.19 | 0.196 |  |  |
|  | I vs. F |  |  | 0.190 | 10 | 5 |
|  | I vs. M |  |  | 0.999 | 10 | 3 |
|  | F vs. M |  |  | 0.386 | 5 | 3 |
| *f*_H_ at 0 bar vs. Activity (0 bar) |  | 0.017 | 0.001 | 0.983 |  |  |
|  | 0 vs. 1 |  |  | 0.987 | 9 | 17 |
|  | 0 vs. 2 |  |  | 0.999 | 9 | 2 |
|  | 1 vs. 2 |  |  | 0.990 | 17 | 2 |
| Unpaired T-Test |  |  |  |  |  |  |
| *f*_H_ at 0 bar vs. Temperature | 10 vs. 12 | 2.16 | 0.18 | **0.018** | 22 | 8 |
| *f*_H_ Scope vs. Temperature | 10 vs. 12 | 2.23 | 0.01 | 0.573 | 20 | 8 |
| Linear Regression |  |  |  |  |  |  |
| *f*_H_ at 0 bar vs. Weight |  | 1.49 | 0.06 | 0.233 | 27 |  |
| *f*_H_ Scope vs. Weight |  | 0.02 | 0.0001 | 0.885 | 27 |  |
| *f*_H_ at 0 bar vs. Length |  | 0.83 | 0.03 | 0.369 | 30 |  |
| *f*_H_ Scope vs. Length |  | 0.06 | 0.002 | 0.812 | 30 |  |
| *f*_H_ Scope vs. Activity (20-80 bar) |  | 0.35 | 0.013 | 0.562 | 28 |  |
| *f*_H_ Scope vs. *f*_H_ at 0 bar |  | 23.5 | 0.47 | **<0.0001** | 30 |  |

Linear regressions, unpaired t-tests, and ANOVAs followed by Tukey’s post-hoc tests performed in Prism examined the effect of sex (I = immature, F = female, M = male), acclimation temperature (10 or 12°C), weight (g), length (cm), initial activity rank at 0 bar, and average activity during pressure exposure (20 to 80 bar) on the initial *f*_H_ at 0 bar and *f*_H_ scope at 0 vs. 80 bar in pressure-exposed lumpfish from the experiments conducted in the IPOCAMP. Significant P values (<0.05) are represented in bold type. *f*_H_ = heart rate.

**Table S5:** Relationships between *f*_H_ and percentage change in *f*_H_ with changes in environmental variables (Exp. ^#^2 and 3).

| Relationship | Treatment | Equation | R^2^ | P |
| --- | --- | --- | --- | --- |
| Decreased Temperature | |  |  |  |
| *f*_H_ x Temperature | Control | Y = 3.49x + 16.15 | 0.92 | **<0.0001** |
|  | Pressure-Exposed | Y = 4.08x + 16.04 | 0.94 | **<0.0001** |
|  | Are the slopes equal? |  |  | **0.007** |
|  | Are the intercepts equal? |  |  | NA |
| % Change in *f*_H_ x Temperature | Control | Y = 5.93x + 27.14 | 0.90 | **<0.0001** |
|  | Pressure-Exposed | Y = 6.01x + 25.32 | 0.93 | **<0.0001** |
|  | Are the slopes equal? |  |  | 0.846 |
|  | Are the intercepts equal? |  |  | 0.1319 |
| Increased Temperature | |  |  |  |
| *f*_H_ x Temperature | Control | Y = 1.53x + 33.17 | 0.54 | **<0.0001** |
|  | Pressure-Exposed | Y = 2.81x + 29.07 | 0.49 | **<0.0001** |
|  | Are the slopes equal? |  |  | **0.0078** |
|  | Are the intercepts equal? |  |  | NA |
| % Change in *f*_H_ x Temperature | Control | Y = 3.34x + 58.73 | 0.53 | **<0.0001** |
|  | Pressure-Exposed | Y = 5.02x + 36.52 | 0.42 | **<0.0001** |
|  | Are the slopes equal? |  |  | 0.1069 |
|  | Are the intercepts equal? |  |  | **0.0324** |
| Decreased Oxygen | |  |  |  |
| *f*_H_ x Temperature | Control | Y = -1.31x + 71.31 | 0.39 | **<0.0001** |
|  | Pressure-Exposed | Y = 0.01x + 63.37 | 0.002 | 0.7859 |
|  | Are the slopes equal? |  |  | **0.0002** |
|  | Are the intercepts equal? |  |  | NA |
| % Change in *f*_H_ x Temperature | Control | Y = -0.30x + 132.4 | 0.48 | **<0.0001** |
|  | Pressure-Exposed | Y = 0.01x + 100.6 | 0.002 | 0.7603 |
|  | Are the slopes equal? |  |  | **<0.0001** |
|  | Are the intercepts equal? |  |  | NA |
| Acute Warming | |  |  |  |
| *f*_H_ x Temperature | Control | Y = 0.29x + 52.83 | 0.02 | **0.0496** |
|  | Warming | Y = 3.41x + 11.16 | 0.50 | **<0.0001** |
|  | Are the slopes equal? |  |  | **<0.0001** |
|  | Are the intercepts equal? |  |  | NA |
| % Change in *f*_H_ x Temperature | Control | Y = 0.56x + 88.85 | 0.02 | **0.037** |
|  | Warming | Y = 6.96x + 16.34 | 0.42 | **<0.0001** |
|  | Are the slopes equal? |  |  | **<0.0001** |
|  | Are the intercepts equal? |  |  | NA |

Relationships between *f*_H_ and percentage change in *f*_H_, and changes in environmental variables (decreased temperature, increased temperature, decreased oxygen, or increased temperature up to 20.8°C in an acute warming experiment), for control and pressure-exposed / warming groups. Significant relationships, and significant differences in slopes or intercepts (P < 0.05) are represented in bold type. *f*_H_ = heart rate.

**Table S6:** Summary of the LME model statistical outputs between ECG quality and changes in environmental variables (Exp. ^#^2 and 3).

| Independent Factor | Dependent Factor | NumDF | DenDF | F-value | P |
| --- | --- | --- | --- | --- | --- |
| Decreased Temperature | |  |  |  |  |
| % of QI_0_ ECGs | (Intercept) | 1 | 83 | 253.0 | **<0.0001** |
|  | Temperature Step | 7 | 83 | 4.6 | **0.0002** |
|  | Treatment | 1 | 12 | 0.0007 | 0.9784 |
|  | Interaction | 7 | 83 | 1.0 | 0.4397 |
| Increased Temperature | |  |  |  |  |
| % of QI_0_ ECGs | (Intercept) | 1 | 90 | 38.7 | **<0.0001** |
|  | Temperature Step | 7 | 90 | 5.5 | **<0.0001** |
|  | Treatment | 1 | 13 | 1.9 | 0.1939 |
|  | Interaction | 7 | 90 | 1.1 | 0.3483 |
| Decreased Oxygen | |  |  |  |  |
| % of QI_0_ ECGs | (Intercept) | 1 | 111 | 46.9 | **<0.0001** |
|  | Oxygen Step | 8 | 111 | 1.3 | 0.2698 |
|  | Treatment | 1 | 14 | 5.9 | **0.0292** |
|  | Interaction | 8 | 111 | 1.9 | 0.0621 |
| Acute Warming | |  |  |  |  |
| % of QI_0_ ECGs | (Intercept) | 1 | 89 | 42.4 | **<0.0001** |
|  | Treatment | 1 | 10 | 13.7 | **0.0041** |
|  | Temperature Step | 9 | 89 | 2.0 | **0.0491** |
|  | Interaction | 9 | 89 | 3.3 | **0.0015** |

LME models examined the effects of treatment (control vs. pressure-exposed), changes in environmental variables (decreased temperature, increased temperature, decreased oxygen or increased temperature in an acute warming experiment), and their interaction, on the percentage of ECGs that were that of ‘good’ quality (i.e., QI_0_). Significant P values (<0.05) are represented in bold type. NumDF = degrees of freedom of the numerator of the F distribution ratio; DenDF = degrees of freedom of the denominator of the F distribution ratio.
